# EHMTI-0368. Treatment of migraine attacks as suggested by Dr. John R. Graham in 1955. A historical analysis with current implications

**DOI:** 10.1186/1129-2377-15-S1-M11

**Published:** 2014-09-18

**Authors:** P Tfelt-Hansen, E Loder

**Affiliations:** 1 Neurology Danish Headache Center, Glostrup Hospital, Glostrup, Denmark; 2 J.R.Graham Headache Center, Harvard Medical School, Boston, USA

## Introduction

In 1955 Dr. John R. Graham, an astute clinician, published 3 influential papers on migraine theories and mainly on treatment of migraine in the New England Journal of Medicine [1,2,3]. Many of Dr. Graham’s clinical observations remain relevant to current methods of treating migraine attacks and some of his examples of suggestions are shown below.

Examples of suggestions on ergot therapy:

1. The patient should be checked regarding to diagnosis of migraine and possible contraindications to ergot therapy.

2. The ergot dose should be carefully selected as the minimally effective dose.

3. The patient should be instructed to use ergot as early in the attack as he can make the diagnosis of “one of his migraine.”

4. He should be urged to use the selected amount of ergot at the start rather than to distribute it over several hours.

5. Ergot derivatives should not be considered a failure until ergotamine tartrate has been given, early in an attack, by the parenteral route.

## Conclusion

If the word “ergot” is replaced with” triptans” Dr. Graham thus seemingly anticipated in 1955 optimal modern acute treatment of migraine.

No conflict of interest.
